# Determinants of site of tuberculosis disease: An analysis of European surveillance data from 2003 to 2014

**DOI:** 10.1371/journal.pone.0186499

**Published:** 2017-11-20

**Authors:** Giovanni Sotgiu, Dennis Falzon, Vahur Hollo, Csaba Ködmön, Nicolas Lefebvre, Andrei Dadu, Marieke van der Werf

**Affiliations:** 1 Clinical Epidemiology and Medical Statistics Unit, Medical Education and Professional Development Unit, Department of Biomedical Sciences, University of Sassari, Sassari, Italy; 2 Global TB Programme, World Health Organization, Geneva, Switzerland; 3 European Centre for Disease Prevention and Control (ECDC), Stockholm, Sweden; 4 Centre Hospitalier Universitaire de Strasbourg, Strasbourg, France; 5 WHO Regional Office for Europe, Copenhagen, Denmark; Hospital San Agustin, SPAIN

## Abstract

**Background:**

We explored host-related factors associated with the site of tuberculosis (TB) disease using variables routinely collected by the 31 EU/EEA countries for national surveillance.

**Methods:**

Logistic regression models were fitted to case-based surveillance data reported to the European Centre for Disease Prevention and Control for TB cases notified from 2003 to 2014. Missing data on HIV infection and on susceptibility to isoniazid and rifampicin for many patients precluded the inclusion of these variables in the analysis. Records from Finland, Lithuania, Spain and the United Kingdom were excluded for lack of exact details of disease localisation; other records without one or more variable (e.g. previous treatment history, geographical origin) or who had mixed pulmonary and extrapulmonary disease or more than one form of extrapulmonary disease were also removed (total exclusion = 38% of 913,637 notifications).

**Results:**

564,916 TB cases reported by 27 EU/EEA countries had exclusive pulmonary (PTB; 83%) or extrapulmonary (EPTB; 17%) disease. EPTB was associated with age <15 years (aOR: 5.50), female sex (aOR: 1.60), no previous TB treatment (aOR: 3.10), and geographic origin (aOR range: 0.52–3.74). Origin from the Indian subcontinent or Africa was most strongly associated with lymphatic, osteo-articular and peritoneal/digestive localization (aOR>3.7), and age <15 years with lymphatic (aOR: 17.96) and central nervous system disease (aOR: 11.41).

**Conclusions:**

Awareness of host-related determinants of site of TB is useful for diagnosis. The predilection for EPTB among patients originating from countries outside Europe may reflect strain preferences for disease localization, geographic/ethnic differences in disease manifestation and other factors, like HIV.

## Introduction

Tuberculosis (TB) remains a serious international public health problem today, with a disproportionate number of the 10 million new cases emerging each year being concentrated in Asia and Africa [1]. The countries of the European Union and European Economic Area (EU/EEA) together reported 58,008 cases in 2014 to the joint TB surveillance activities of the European Centre for Disease Prevention and Control (ECDC) and the World Health Organisation (WHO) (Table 1), which account for about 1% of all TB cases notified globally [2]. Despite its relatively small and declining TB caseload, this group of 31 countries presents a very diverse TB epidemiological pattern, with low TB incidence in its western and southern regions to moderate levels moving eastwards. The large majority of TB cases in western European countries are of foreign origin or in subgroups at a higher risk of infection and disease than the general population. Overall, 75% of the TB cases show exclusive pulmonary involvement (PTB), 19% solely extrapulmonary disease (EPTB), and 6% have mixed disease [3]. These proportions, however, differ markedly between countries and population groups. In general, PTB patients have more severe forms of disease, as evidenced by a higher risk of dying and lower risk of completing treatment successfully when compared to the average EPTB case. Given this unfavourable prognosis, as well as the risk of direct transmissibility, PTB remains a priority for public health action in TB control. Nonetheless, EPTB also presents public health concerns given that it often challenges diagnosis (particularly in children), and it could have clinically significant sequelae [4]. A number of high-income countries have reported an increased frequency of EPTB over time [3,5,6]. Site of disease has been reported to differ substantially by geography and by other risk factors [3, 7–10].

**Table 1:** Demographic and clinical characteristics of tuberculosis cases notified in 31 European Union and European Economic Area countries, by site of disease, 2003–2014.

Characteristics	Sites								Total	
									(N = 913,637)	
	Pulmonary only		Extrapulmonary only		Both sites		Site unknown			
	(N = 630,356)		(N = 179,357)		(N = 56,676)		(N = 47,248)			
	N	%	N	%	N	%	N	%	N	%
**Year of report**										
2003	51937	8%	14184	8%	5593	10%	14882	31%	86596	9%
2004	60368	10%	14914	8%	5206	9%	3287	7%	83775	9%
2005	58903	9%	14428	8%	4965	9%	2419	5%	80715	9%
2006	55314	9%	14724	8%	4554	8%	1447	3%	76039	8%
2007	56520	9%	14566	8%	4900	9%	8033	17%	84019	9%
2008	54502	9%	15317	9%	4836	9%	8429	18%	83084	9%
2009	52089	8%	15353	9%	4440	8%	7770	16%	79652	9%
2010	53894	9%	16863	9%	4628	8%	190	0%	75575	8%
2011	51290	8%	16056	9%	4784	8%	233	0%	72363	8%
2012	48720	8%	15806	9%	4350	8%	114	0%	68990	8%
2013	45743	7%	14468	8%	4438	8%	172	0%	64821	7%
2014	41076	7%	12678	7%	3982	7%	272	1%	58008	6%
**Age group** (years)										
< 15	13946	2%	14075	8%	6208	11%	2880	6%	37109	4%
15–44	289339	46%	89865	50%	27943	49%	22687	48%	429834	47%
45–64	211789	34%	40790	23%	12731	22%	11834	25%	277144	30%
≥ 65	113985	18%	34304	19%	9726	17%	9544	20%	167559	18%
Unknown	1297	0%	323	0%	68	0%	303	1%	1991	0%
**Sex**										
Female	203080	32%	82761	46%	20628	36%	17455	37%	323924	35%
Male	426530	68%	96309	54%	35939	63%	29414	62%	588192	64%
Unknown	746	0%	287	0%	109	0%	379	1%	1521	0%
Previous TB treatment*										
No	489667	78%	152501	85%	47109	83%	39356	83%	728633	80%
Yes	99982	16%	7478	4%	3630	6%	3308	7%	114398	13%
Unknown	40707	6%	19378	11%	5937	10%	4584	10%	70606	8%
Region of origin**										
Western Europe	114653	18%	39764	22%	10876	19%	3799	8%	169092	19%
Central Europe, EU since 2004	400434	64%	60371	34%	22663	40%	1987	4%	485455	53%
Other Central / Eastern Europe	10787	2%	1730	1%	861	2%	576	1%	13954	2%
Africa	25133	4%	21641	12%	6185	11%	2219	5%	55178	6%
Indian subcontinent	14300	2%	24573	14%	5052	9%	647	1%	44572	5%
Rest of Asia	15835	3%	8914	5%	2642	5%	1137	2%	28528	3%
Americas & Oceania	4911	1%	2163	1%	570	1%	377	1%	8021	1%
Unknown	44303	7%	20201	11%	7827	14%	36506	77%	108837	12%
**Country of report**										
Austria	7074	1%	1732	1%	798	1%	4	0%	9608	1%
Belgium	7822	1%	3529	2%	1193	2%	2	0%	12546	1%
Bulgaria	14453	2%	4980	3%	803	1%	1	0%	20237	2%
Croatia	1318	0%	140	0%	58	0%	73	0%	1589	0%
Cyprus	406	0%	121	0%	23	0%	2	0%	552	0%
Czech Republic	7428	1%	1561	1%	445	1%	0	0%	9434	1%
Denmark	2557	0%	824	0%	166	0%	963	2%	4510	0%
Estonia	4211	1%	401	0%	423	1%	0	0%	5035	1%
Finland	2742	0%	1171	1%	0	0%	0	0%	3913	0%
France	37482	6%	16630	9%	8817	16%	915	2%	63844	7%
Germany	41348	7%	13186	7%	5629	10%	623	1%	60786	7%
Greece	3447	1%	601	0%	399	1%	2882	6%	7329	1%
Hungary	18479	3%	1036	1%	246	0%	0	0%	19761	2%
Iceland	59	0%	54	0%	18	0%	0	0%	131	0%
Ireland	3124	0%	1520	1%	397	1%	21	0%	5062	1%
Italy	31387	5%	11898	7%	1862	3%	1	0%	45148	5%
Latvia	10270	2%	1260	1%	631	1%	1726	4%	13887	2%
Liechtenstein	4	0%	1	0%	0	0%	0	0%	5	0%
Lithuania	23017	4%	3118	2%	7	0%	0	0%	26142	3%
Luxembourg	334	0%	45	0%	3	0%	29	0%	411	0%
Malta	250	0%	122	0%	46	0%	1	0%	419	0%
Netherlands	4505	1%	4347	2%	1166	2%	2672	6%	12690	1%
Norway	2083	0%	1431	1%	360	1%	106	0%	3980	0%
Poland	82639	13%	6579	4%	530	1%	10124	21%	99872	11%
Portugal	24503	4%	8624	5%	2285	4%	1175	2%	36587	4%
Romania	224583	36%	38813	22%	18278	32%	17	0%	281691	31%
Slovakia	5282	1%	1255	1%	382	1%	0	0%	6919	1%
Slovenia	1680	0%	408	0%	364	1%	2	0%	2454	0%
Spain	22399	4%	8344	5%	0	0%	23576	50%	54319	6%
Sweden	2332	0%	1965	1%	527	1%	1925	4%	6749	1%
United Kingdom	43138	7%	43661	24%	10820	19%	408	1%	98027	11%
**HIV infection**										
Positive	3866	1%	1511	1%	816	1%	7	0%	6200	1%
Negative	84890	13%	18082	10%	5148	9%	125	0%	108245	12%
Unknown	541600	86%	159764	89%	50712	89%	47116	100%	799192	87%
**Culture result**										
Positive	434638	69%	59974	33%	35075	62%	24633	52%	554320	61%
Negative	118063	19%	31452	18%	11391	20%	8863	19%	169769	19%
Unknown	77655	12%	87931	49%	10210	18%	13752	29%	189548	21%
Multidrug resistance***										
Yes	14609	4%	563	1%	558	2%	213	2%	15943	4%
No	205256	57%	40821	77%	20033	72%	4742	51%	270852	60%
Unknown	139845	39%	11651	22%	7167	26%	4345	47%	163008	36%

* Cases without a previous TB diagnosis but with no information on treatment history were considered new cases;

** Western Europe includes European countries not included in Central or Eastern Europe;

Central Europe, EU since 2004 is composed of Bulgaria, Croatia, Czech Rep, Estonia, Hungary, Latvia, Lithuania, Poland, Romania, Slovenia and Slovakia;

Other Central / Eastern Europe includes all other Central European countries in the Balkans as well as Belarus, Moldova Rep., the Russian Federation and Ukraine;

The Indian subcontinent is composed of Bangladesh, Bhutan, British Indian Ocean Territory, Comoros Islands, India, Maldives, Nepal, Pakistan and Sri Lanka;

Rest of Asia includes the former Soviet Central Asian republics, and other Asian countries not in the Indian subcontinent or Oceania.

*** Resistance to isoniazid and rifampicin in cases with a strain reported as M.tuberculosis complex, M.tuberculosis, M.bovis, or M.africanum

In the EU/EEA, the reporting of case-based data for the European-level surveillance of TB dates back to the mid-1990s. These data allow an in-depth study of epidemiological patterns and determinants of outcomes (e.g. drug resistance, death). In this paper, we analyse data reported by various EU/EEA countries in the 12 most recent years to identify demographic and clinical host-related factors associated with the site of disease among TB cases.

## Materials & methods

### Data sources and collection

Surveillance data described in this article were reported within the framework of collaborative surveillance of TB in Europe through a network of national TB surveillance authorities [11,12]. Reporting has followed a standardised methodology allowing comparison between countries and over time [13–16]. Anonymous case-based data are now consolidated as part of the European Surveillance System (or TESSy) by the European Centre for Disease Prevention and Control (ECDC; www.ecdc.europa.eu), based in Sweden. Until 2007, the European TB data collection was coordinated by a dedicated network (EuroTB) coordinated by the French Institut de Veille Sanitaire, a WHO Collaborating Centre.

### Inclusion criteria and definitions

**Fig 1:**
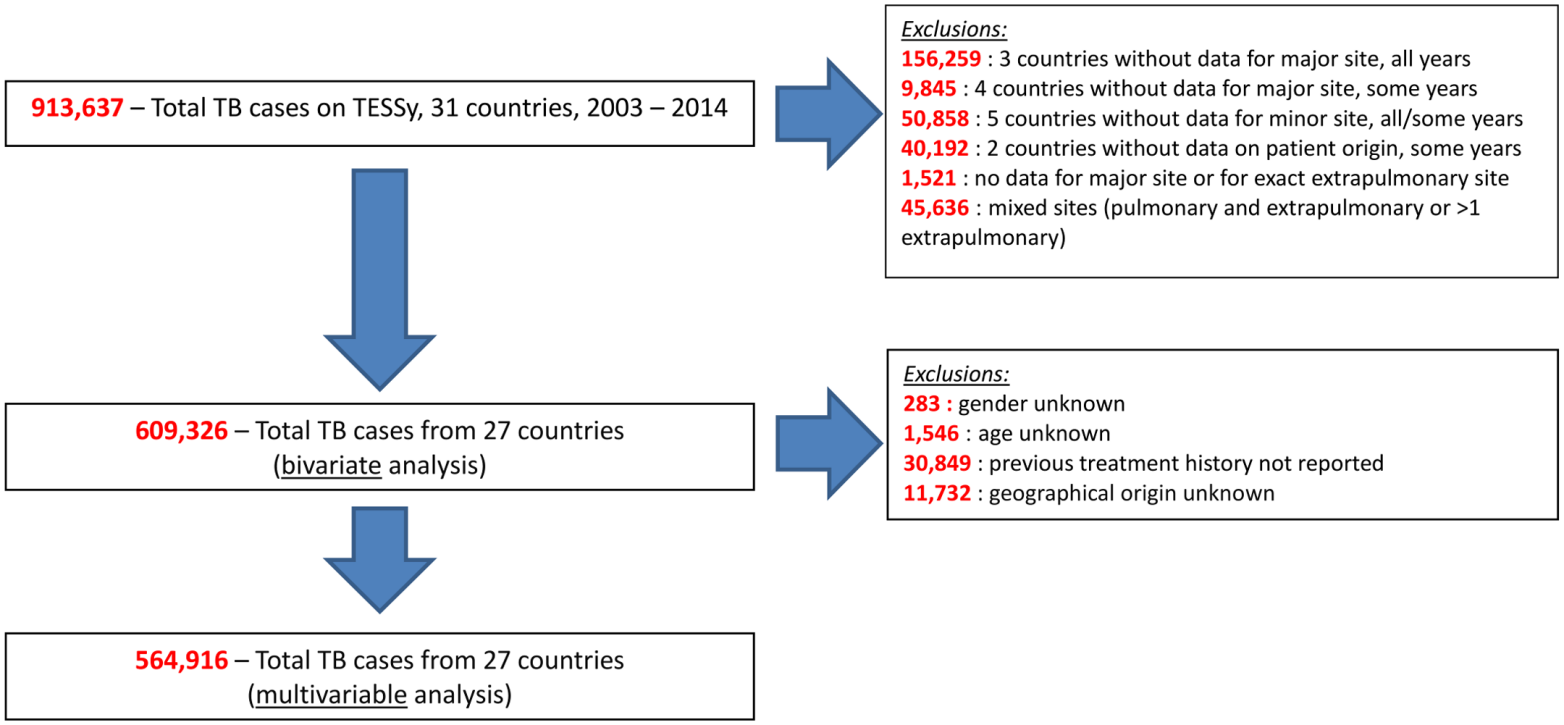
Selection of records for the analysis of determinants of site of TB, EU/EEA countries, 2003–2014.

For this analysis, PTB is defined as TB of the lung parenchyma, tracheo-bronchial tree, or larynx, while EPTB refers to TB affecting any other anatomical site. The preferred method of reporting site of disease requires information on both the major and minor localisation, as well as details of the specific site of EPTB disease. This method allows reporting of up to two sites; if more than two sites are present in an individual patient, only the two main sites are included. The following groupings of EPTB cases were used: pleural, lymphatic (both intra-thoracic and extra-thoracic), osteo-articular (spine, bone, and joint), central nervous system (CNS; including meningeal site), genito-urinary (kidney, ureter, bladder, genital tract), peritoneal/digestive tract and disseminated (including TB of >two organ systems, miliary TB or the isolation of *Mycobacterium tuberculosis* from the blood). In order to explore determinants of specific disease localisation, only patients with either exclusive PTB or exclusive EPTB were retained in the analysis (45,636 exclusions made for patients with both PTB and EPTB involvement or with two EPTB sites; Fig 1).

**Fig 2:**
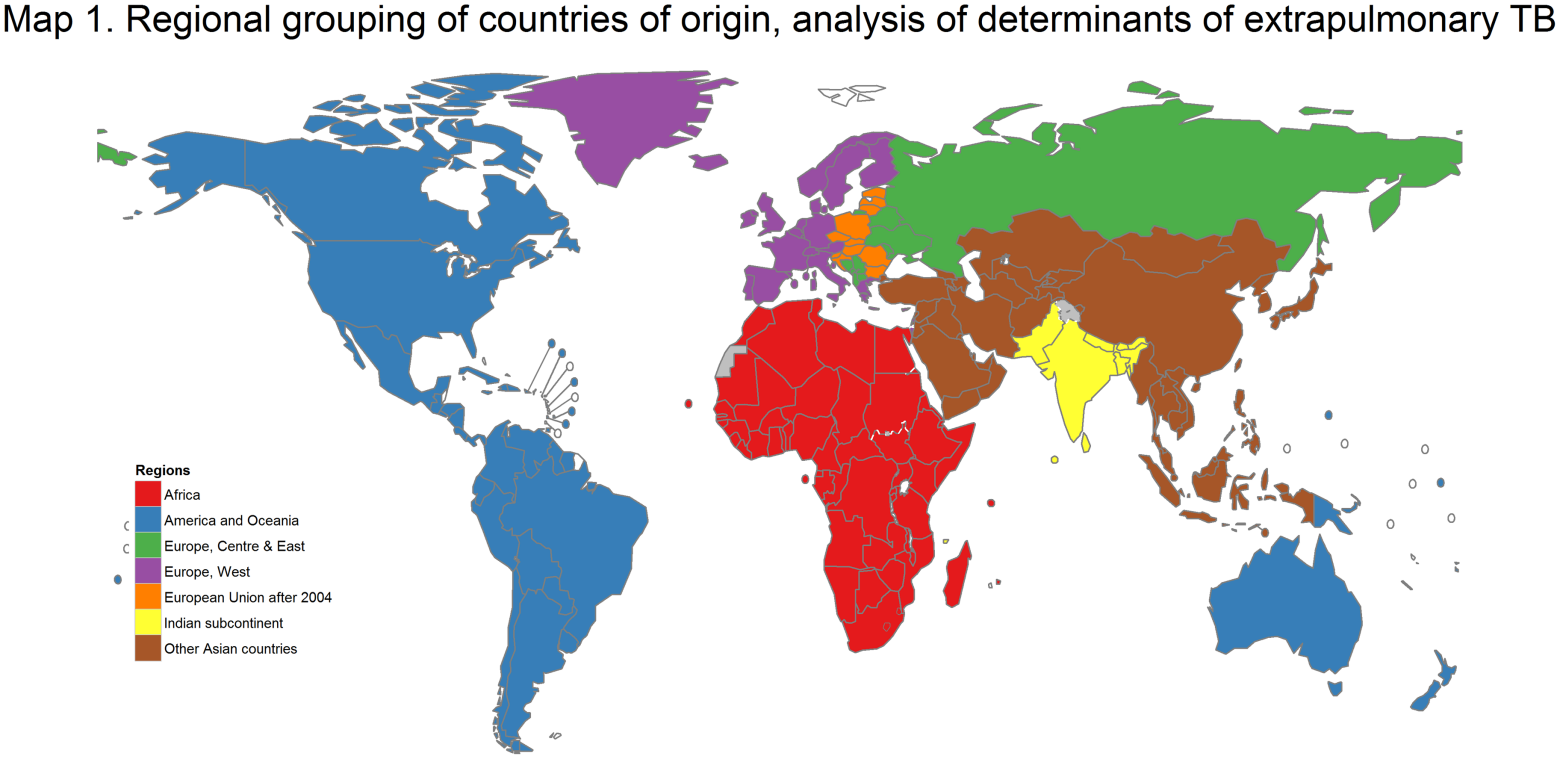
Regional grouping of countries of origin used in the analysis of determinants of site of TB, EU/EEA countries, 2003–2014. The boundaries and names shown and the designations used on this map do not imply the expression of any opinion whatsoever on the part of the World Health Organization concerning the legal status of any country, territory, city or area or of its authorities, or concerning the delimitation of its frontiers or boundaries. Dotted lines on maps represent approximate border lines for which there may not yet be full agreement.

Country of origin was defined by birth in all included countries except for Austria and Belgium where it was defined by citizenship. Countries were classified in seven geographical groups, namely Africa, Americas & Oceania, Europe (three subgroupings), the Indian subcontinent, and the Rest of Asia (Fig 2; Table 1 footnote). The three European subgroupings were: Central European countries which joined the EU since 2004 (Bulgaria, Croatia, Czech Republic, Estonia, Hungary, Latvia, Lithuania, Poland, Romania, Slovakia, and Slovenia); other Central and Eastern Europe (Belarus, Republic of Moldova, the Russian Federation and Ukraine as well as all other Central European countries in the Balkans); and Western Europe (the remaining countries). The Indian subcontinent grouped the following Asian countries: Bangladesh, Bhutan, India, Maldives, Nepal, Pakistan and Sri Lanka. Rest of Asia consisted of other countries in the region not in the Indian subcontinent or Oceania, and includes Central Asia (Fig 2).

Children were defined as individuals who were under 15 years of age at the time of the report; elderly were those over 64 years. Previous TB treatment referred to chemotherapy given for active TB disease for more than four weeks and started in a calendar year before the year of report. Cases without a previous TB diagnosis and with no information on treatment history were considered new cases. Culture confirmation referred to the isolation of *M*. *tuberculosis* complex. Multidrug resistance (MDR-TB) was defined as *in vitro* resistance to at least isoniazid and rifampicin in a strain reported as *M*. *tuberculosis* complex, *M*. *tuberculosis*, *M*. *bovis*, or *M*. *africanum* on tissue samples taken at the start of treatment.

The study period consisted of the 12 consecutive years from 2003 to 2014 inclusive. Countries eligible for inclusion in this analysis were the 28 belonging to the EU as in 2014, as well as Iceland, Liechtenstein and Norway (Table 1). Countries which did not report complete details of EPTB localisation were not included in the analysis (Finland, Lithuania, Spain and the United Kingdom); records from other countries were excluded for earlier years when no data were reported for site of disease (Denmark, Latvia, Poland and Sweden) or patient geographic origin (France, Norway and Poland) (Fig 1).

### Statistical analysis

The ‘null hypothesis’ of no association between site and putative explanatory variables was tested using data from all countries pooled for all available years. Bivariate and multivariable analyses were restricted to cases with full data on site, sex, age, TB treatment history, and geographic origin. Owing to the large proportion of cases without information on (i) susceptibility to isoniazid and rifampicin and (ii) HIV status (Table 1), the analysis did not consider these variables.

Crude odds ratios (OR) with 95% confidence intervals (95% CI) were used to express magnitude of association between categorical variables in bivariate analysis (Table 2). Pearson’s χ2 was used to test relationships and a p-value<0.05 was considered statistically significant and was also used as the threshold to include variables in the logistic regression (see below).

**Table 2:** Bivariate and multivariable (logistic regression) analysis for risk factors associated with exclusive extrapulmonary tuberculosis in 27 European Union and European Economic Area countries^1^, 2003–2014 (N = 564,916 cases with full data).

Risk factors	Sites						Bivariate		Multivariable	
	Pulmonary only		Extrapulmonary only							
	(N = 469,913)		(N = 95,003)		Total					
					(N = 564,916)					
	N	%	N	%	N	%	OR	95%CI	aOR	95%CI
**Age group** (years)										
< 15	7966	2%	9417	10%	17383	3%	5.84	5.64–6.03	5.50	5.32–5.68
15–44	213019	45%	43112	45%	256131	45%	1	reference	1.00	reference
45–64	168373	36%	23042	24%	191415	34%	0.68	0.66–0.69	0.88	0.86–0.89
≥ 65	80555	17%	19432	20%	99987	18%	1.19	1.17–1.21	1.26	1.24–1.29
**Sex**										
Female	146301	31%	43296	46%	189597	34%	1.85	1.82–1.88	1.60	1.58–1.63
Male	323612	69%	51707	54%	375319	66%	1	reference	1	reference
**Previous TB treatment**^2^										
No	381915	81%	90155	95%	472070	84%	1	reference	1	reference
Yes	87998	19%	4848	5%	92846	16%	0.23	0.23–0.24	0.32	0.31–0.33
**Region of origin**										
Western Europe	68131	14%	21447	23%	89578	16%	1	reference	1	reference
Central Europe, EU since 2004	364087	77%	55116	58%	419203	74%	0.48	0.47–0.49	0.57	0.56–0.58
Other Central / Eastern Europe	8541	2%	1339	1%	9880	2%	0.50	0.47–0.53	0.52	0.49–0.56
Africa	12607	3%	8291	9%	20898	4%	2.09	2.02–2.16	2.29	2.22–2.38
Indian subcontinent	3300	1%	3377	4%	6677	1%	3.25	3.09–3.42	3.74	3.56–3.94
Rest of Asia	10611	2%	4547	5%	15158	3%	1.36	1.31–1.41	1.47	1.42–1.53
Americas & Oceania	2636	1%	886	1%	3522	1%	1.07	0.99–1.15	1.08	0.99–1.16

EU, European Union; OR, Odds ratio; aOR, adjusted OR; 95%CI, 95% confidence limits

^1^ Including countries listed in Table 1 with the exception of Finland, Lithuania, Spain, and the United Kingdom (see Fig 2 and Materials and methods for more detail on exclusion of data)

^2^ Cases without a previous TB diagnosis and with no information on treatment history were considered new cases

Two multivariable regression analyses were performed. Binary logistic regression was first used to identify variables independently associated with exclusive EPTB disease (Table 2). Multinomial logistic regression was used to compare the risk factors for different forms of EPTB, using PTB as a reference (Table 3). To simplify the presentation, the results of multinomial regression are shown without adjustment for interaction. Adjusted ORs (aORs) were used to quantify the magnitude of associations.

**Table 3:** Risk factors associated with different forms of extrapulmonary tuberculosis (N = 95,003) compared to pulmonary tuberculosis (N = 469,913) in 27 countries of the European Union and European Economic Area, 2003–2014.

Risk factors	Pleural		Lymphatic		Osteo-articular		Genito-urinary		Central nervous system		Peritoneal & Digestive		Disseminated		Other	
	(N = 38,026)		(N = 28,019)		(N = 8,219)		(N = 6,008)		(N = 3,180)		(N = 2,780)		(N = 1,190)		(N = 7,581)	
	aOR	95%CI	aOR	95%CI	aOR	95%CI	aOR	95%CI	aOR	95%CI	aOR	95%CI	aOR	95%CI	aOR	95%CI
**Age group** (years)																
< 15	1.93	1.83–2.04	17.96	17.21–18.74	4.48	4–5.02	0.53	0.37–0.75	11.41	10.29–12.65	2.21	1.8–2.73	2.58	1.93–3.43	3.73	3.32–4.18
15–44	1	reference	1	reference	1	reference	1	reference	1	reference	1	reference	1	reference	1	reference
45–64	0.57	0.55–0.58	0.93	0.9–0.97	1.95	1.85–2.07	2.43	2.28–2.6	0.96	0.87–1.05	1.03	0.94–1.13	0.88	0.75–1.02	1.62	1.53–1.72
≥ 65	0.75	0.72–0.77	1.52	1.46–1.57	3.33	3.14–3.53	3.35	3.12–3.59	1.07	0.96–1.19	1.25	1.12–1.39	1.24	1.06–1.45	2.39	2.25–2.54
Female sex	1.08	1.06–1.11	2.55	2.49–2.62	1.63	1.56–1.7	1.78	1.69–1.87	1.58	1.47–1.7	2.02	1.87–2.18	1.23	1.09–1.39	2.31	2.2–2.42
Previous TB treatment	0.15	0.14–0.15	0.48	0.45–0.5	0.59	0.55–0.63	0.45	0.41–0.5	0.42	0.37–0.49	0.45	0.38–0.52	0.54	0.43–0.69	0.52	0.48–0.57
**Region of origin**																
Western Europe	1	reference	1	reference	1	reference	1	reference	1	reference	1	reference	1	reference	1	reference
Central Europe, EU since 2004	1.08	1.05–1.11	0.37	0.36–0.38	0.53	0.5–0.56	0.3	0.28–0.32	0.55	0.51–0.6	0.36	0.33–0.4	0.09	0.08–0.1	0.31	0.3–0.33
Other Central / Eastern Europe	0.49	0.44–0.54	0.65	0.59–0.72	0.59	0.49–0.71	0.59	0.5–0.7	0.59	0.45–0.78	0.41	0.29–0.59	0.29	0.19–0.46	0.29	0.23–0.36
Africa	0.86	0.8–0.92	4.65	4.43–4.88	3.83	3.51–4.17	1.06	0.93–1.21	1.88	1.61–2.19	4.64	4.1–5.25	2.21	1.86–2.61	2.57	2.36–2.8
Indian subcontinent	1.23	1.1–1.37	8.84	8.27–9.45	6.83	6.06–7.7	1.23	0.97–1.55	2.46	1.93–3.15	6.36	5.33–7.58	2.97	2.31–3.81	3.72	3.27–4.24
Rest of Asia	0.59	0.54–0.65	3.22	3.06–3.4	1.48	1.31–1.66	0.9	0.79–1.03	0.96	0.77–1.18	2.07	1.77–2.43	0.5	0.36–0.68	1.09	0.97–1.23
Americas & Oceania	0.72	0.62–0.84	1.69	1.51–1.9	1.12	0.86–1.46	0.57	0.41–0.81	1	0.68–1.46	2.22	1.69–2.91	0.78	0.48–1.27	1.26	1.03–1.55

aOR, Adjusted Odds Ratios from multinomial regression analysis; EU, European Union; 95% CI, 95% confidence limits

Values not adjusted for clustering by country of report or for year of report

Maps were created with R (using *ggplot2* package) working within R Studio [17]. Logistic regression was done with R (*glm* function) and multinomial regression with STATA version 12.1 (StataCorp, College Station, Texas).

## Results

### Description

The thirty-one EU/EEA countries reported a total of 913,637 TB cases, fairly evenly spread over the 12-year period (Table 1). Half the included cases were from Romania (31%), Poland (11%) and the United Kingdom (11%). Out of all cases reported, 69% were exclusively PTB, 20% were exclusively EPTB, 6% had both PTB and EPTB, and 5% had an unknown site. Culture confirmation was more frequent in PTB (69%) than in exclusive EPTB cases (33%), half of whom had no culture result. Multidrug resistance was confirmed in 4% of PTB cases and 1% of EPTB, with 36% overall having no result. HIV infection was recorded in 1% overall but 87% of cases had no information.

**Fig 3:**
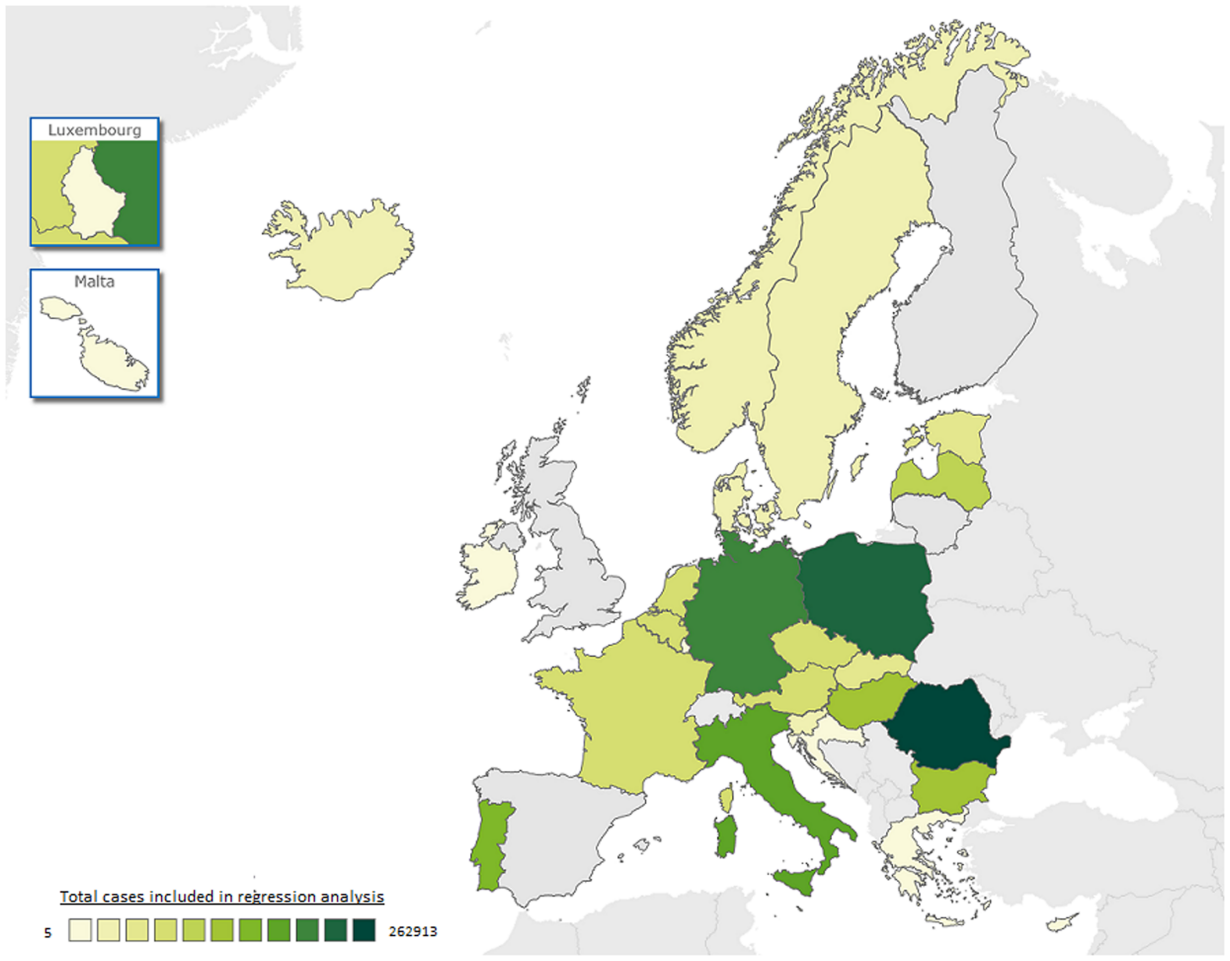
Distribution of TB cases in the 27 countries included in the multivariable analysis of determinants of site of TB, EU/EEA countries, 2003–2014. SOURCE OF GRAPHIC: ECDC Map Maker (EMMa), available at https://emma.ecdc.europa.eu The boundaries and names shown and the designations used on this map do not imply the expression of any opinion whatsoever on the part of the World Health Organization concerning the legal status of any country, territory, city or area or of its authorities, or concerning the delimitation of its frontiers or boundaries. Dotted lines on maps represent approximate border lines for which there may not yet be full agreement.

After exclusion of cases without key data, 564,916 records (62%; Table 2; Figs 1 and 3) from twenty-seven countries were retained. The characteristics of the cases for the variables of interest had a similar distribution to the one of the whole population before restriction; the only exception is in the proportion of cases from the Central European EU countries (53% in complete dataset and 74% after exclusions; Tables 1 and 2). Seventeen percent of cases had exclusively EPTB. Children and the elderly accounted for smaller proportions of the PTB (2% and 17% respectively) when compared with EPTB cases (10% and 20%). The ratio of males to females was 1.2 among the EPTB cases, but was 2.2 among the PTB cases. More of the EPTB cases were previously untreated for TB (95%) than PTB cases (81%). Only 6% of PTB cases originated from outside Europe, with most reported from Romania and other Central European EU countries. In contrast, a larger proportion of EPTB cases originated from Western Europe (23%), Africa (9%) and Asia (8%). Among the 95,003 exclusively EPTB cases, the most frequent localizations reported were pleural (40%), lymphatic (29%), osteoarticular (9%), and genito-urinary (6%) (Table 3). The more severe forms of EPTB—CNS (3%) and disseminated (1%)—were relatively uncommon.

### Analysis

At bivariate analysis, the strongest, statistically significant positive associations with EPTB were observed with being a child (OR = 5.84), not previously treated for TB (4.28; 0.23 if previously treated), being from Africa (2.09 compared with Western Europe) or the Indian subcontinent (3.25), or female (1.85) (Table 2). The adjusted ORs from logistic regression reproduced the magnitude and direction of associations observed at bivariate analysis. Year of report bore no relationship to the site of disease.

At multinomial regression (Table 3), an origin from the Indian subcontinent or Africa was most strongly associated with lymphatic, osteoarticular and peritoneal/digestive localization (aOR at least 3.83). Age <15 years was most strongly associated with lymphatic and central nervous system disease (aOR at least 11.41), but all types of EPTB were more likely in childhood, with the exception of genito-urinary TB which was associated with age 45–64 years and >64 years (aORs 2.43 and 3.35 respectively). Female sex was significantly associated with all forms of EPTB, especially lymphatic forms (2.55). Patients with a previous history of TB treatment were less likely to have EPTB, regardless of form (aORs ranging from 0.15 in pleural to 0.59 in osteoarticular).

## Discussion

We explored pooled, case-based surveillance data reported by EU/EEA countries for twelve recent years to identify factors associated with the site of disease in notified TB patients. We observed that EPTB was more likely at the extremes of age, in females, in individuals not previously treated for TB, and in patients who were of African or Asian ethnicity. These findings are important for the post-2015 End TB Strategy with its stress on treating all forms of TB [18].

Our analysis quantifies the differential effects of demographic and clinical factors on the disease manifestation using a multinational routine surveillance database which has been consolidated over several years through the long-standing commitment of a vigorous network of public health workers [11]. Two important strengths of the dataset we used were its diversity in terms of patient mix in the countries of the EU/EEA and its size—more than half a million cases from 27 countries could be included in the main analysis. This allowed, for instance, a grouping of patients into geographical subsets in which meaningful patterns of TB localization could be discerned. These included regions of the world from where such information is otherwise sparse.

The effect of age and sex on site of TB disease that we report concurs with what has been described from various settings, albeit with a much larger geographic span and number of observations [19–26]. In our study, the risk for most types of EPTB was associated with the extremes of age (<15 and >64 years), possibly reflecting the performance of an immune system which is still maturing in infancy and childhood or declining with advancing age. The associations we have described between EPTB and female sex have been reported in different parts of the world [9,27,28]. They may be influenced in part by a higher frequency of smoking in males, which is known to predispose to PTB [27].

Our analysis has shown a clear association between certain forms of EPTB and the geographic origin of patients. After adjustment for age, sex, and TB treatment history, cases originating from India, Africa, and Asia were more likely to develop EPTB than cases originating from Europe and Americas, with aORs ranging between 1.5 and 3.7. For lymphatic, osteoarticular and peritoneal/digestive EPTB, the association with an origin from the Indian subcontinent was the highest of all regional variations observed, ranging from 6.4 to 8.8. The association between EPTB and ethnicity was demonstrated in other studies, suggesting a relationship between the genetic background of the host and some *M*. *tuberculosis* genotypes which are more prevalent in some geographical areas [29,30]. Host predisposition for TB and for certain forms of disease based on human genetic characteristics have been described, although the relationship with any particular site was not strong [31,32].

In the dataset we analysed, severe forms of EPTB (CNS and disseminated) were relatively uncommon. Challenges remain to diagnose EPTB, including in well-resourced, high-income settings where TB is becoming increasingly rare [33]. Delays in diagnosis and treatment of EPTB predispose to progression of disease. Maintaining a higher index of suspicion thus remains important, bearing in mind the protean manifestations of TB. The use of appropriate diagnostics is key, particularly molecular techniques which are now validated for the diagnosis of several forms of EPTB and for use in children [34,35].

Some limitations in our analysis should be highlighted. The authors had no means of independently verifying the quality of reporting and any differences in the validity of report between countries and over time. However, most institutions belonging to this European network have been using a fairly standardised reporting methodology for many years. The variables in the ECDC TESSy database do not include those for certain risk factors that are known to influence progression and form of TB disease, such as the delay to start of treatment, adequacy of treatment administered, diabetes, cancer, and other comorbidities, immunosuppressive therapy tobacco smoking, and alcohol consumption [20,21,36–39]. Moreover, incomplete information on susceptibility to isoniazid and rifampicin and on HIV status precluded the investigation of their potential associations with TB localisation within the scope of our analysis. While it may be difficult to harmonise variables about modifiable risk factors like alcohol use and smoking within multinational databases such as TESSy, it may be feasible to do so at national level and explore associations which could guide frontline staff in their work. Finally, it may be useful in the future to use TB strain genotype data being collated within TESSy to explore transmission at the molecular level.

In conclusion, it is likely that the predilection for TB site of disease is determined by a complex interplay between the microbiology and host genetic, behavioural, clinical and demographic features [40,41]. Our analysis sheds light on the determinants of TB localisation from a surveillance dataset spanning a broad swathe of the global population, extending well beyond the high- and middle-income settings of the western and central European countries reporting the data. The fact that our findings correspond to those described elsewhere attests to the utility of routinely collected data for planning and decision-making. It is likely that EPTB will remain a topic of discussion in TB care and prevention, especially given that issues such as early case finding, childhood TB, improved diagnostics, and more effective treatment for all forms of TB remain integral to the implementation of the post-2015 End TB Strategy and to TB elimination in low-incidence settings [18,42].
